# Increase in body weight over a two-year period is associated with an increase in midfoot pressure and foot pain

**DOI:** 10.1186/s13047-017-0214-5

**Published:** 2017-07-25

**Authors:** Tom P. Walsh, Paul A. Butterworth, Donna M. Urquhart, Flavia M. Cicuttini, Karl B. Landorf, Anita E. Wluka, E. Michael Shanahan, Hylton B. Menz

**Affiliations:** 1 College of Medicine and Public Health, Flinders University, Bedford Park, South Australia Australia; 2 Department of Orthopaedics and Trauma, The Queen Elizabeth Hospital, Woodville South, South Australia Australia; 3 Discipline of Podiatry and La Trobe Sport and Exercise Medicine Research Centre, School of Allied Health, College of Science, Health and Engineering, La Trobe University, Bundoora, Victoria Australia; 4 School of Health and Human Sciences, Southern Cross University, Bilinga, Queensland Australia; 5 Department of Epidemiology and Preventive Medicine, School of Public Health and Preventive Medicine, Monash University, Melbourne, Victoria Australia; 6 Department of Rheumatology, Southern Adelaide Local Health Network, Bedford Park, South Australia Australia

**Keywords:** Foot, Pain, Obesity, Kinematics

## Abstract

**Background:**

There is a well-recognised relationship between body weight, plantar pressures and foot pain, but the temporal association between these factors is unknown. The aim of this study was to investigate the relationships between increasing weight, plantar pressures and foot pain over a two-year period.

**Methods:**

Fifty-one participants (33 women and 18 men) completed the two-year longitudinal cohort study. The sample had a mean (standard deviation (SD)) age of 52.6 (8.5) years. At baseline and follow-up, participants completed the Manchester Foot Pain and Disability Index questionnaire, and underwent anthropometric measures, including body weight, body mass index, and dynamic plantar pressures. Within-group analyses examined differences in body weight, foot pain and plantar pressures between baseline and follow up, and multivariate regression analysis examined associations between change in body weight, foot pain and plantar pressure. Path analysis assessed the total impact of both the direct and indirect effects of change in body weight on plantar pressure and pain variables.

**Results:**

Mean (SD) body weight increased from 80.3 (19.3), to 82.3 (20.6) kg, *p* = 0.016 from baseline to follow up. The change in body weight ranged from −16.1 to 12.7 kg. The heel was the only site to exhibit increased peak plantar pressures between baseline and follow up. After adjustment for age, gender and change in contact time (where appropriate), there were significant associations between: (i) change in body weight and changes in midfoot plantar pressure (*B* = 4.648, *p* = 0.038) and functional limitation (*B* = 0.409, *p* = 0.010), (ii) plantar pressure change in the heel and both functional limitation (*B =* 4.054, *p* = 0.013) and pain intensity (*B =* 1.831, *p* = 0.006), (iii) plantar pressure change in the midfoot and both functional limitation (*B =* 4.505*, p* = 0.018) and pain intensity (*B =* 1.913*, p* = 0.015)*.* Path analysis indicated that the effect of increasing body weight on foot-related functional limitation and foot pain intensity may be mediated by increased plantar pressure in the midfoot.

**Conclusions:**

These findings suggest that as body weight and plantar pressure increase, foot pain increases, and that the midfoot may be the most vulnerable site for pressure-related pain.

## Background

Foot pain is common in the community. Approximately one quarter of adults report frequent foot pain [1] and one in six adults aged greater than 50 years experience symptomatic foot osteoarthritis [2]. Foot pain is also associated with pain in other joints, reduced health-related quality of life and obesity [3]. A recent systematic review found that obesity, defined by elevated body mass index (BMI), was strongly associated with chronic plantar heel pain in a non-athletic population and with non-specific foot pain in the general population [4]. Elevated BMI has also been associated with worsening foot pain over a five-year period in women, even after adjusting for age, rheumatoid arthritis and diabetes [5].

One of the mechanisms that may link increased body weight and foot pain is mechanical loading. Increased body mass is known to contribute to elevated peak plantar pressures [6] and elevated peak plantar pressures are associated with foot pain [7]. A recent study of older people found higher midfoot peak pressures and overall foot pain with increased BMI [8]. It seems intuitive, then, that as body weight increases, plantar pressure increases, overloading plantar tissue and causing pain. Furthermore, a previous study has found that midfoot osteoarthritis is associated with higher midfoot pressures, suggestive of a mechanical relationship [9]. Other factors, however, linking foot pain and body mass, such as metabolic and psychological factors have been investigated [10], but whether there is mediation via mechanical pathways is not known.

Indeed, despite this proposed relationship between body weight, plantar pressure and foot pain, previous studies have been cross-sectional and therefore have provided no information regarding the temporal relationship between these factors. This is important, as it is unknown if the foot can adapt to increased body weight over time. As such, the effect of increased body weight on plantar pressures and foot pain may depend on the extent to which the foot can adapt to these changes. Prospective studies are needed to determine if a change in body weight is associated with pathological foot mechanics.

Therefore, the aims of this study were to: (i) examine if a change in body weight is associated with a change in plantar pressures, and to (ii) examine whether a change in body weight and plantar pressures are associated with a change in foot pain intensity or foot-related functional limitation over a two-year period.

## Methods

### Participants

Participants from a previous study [6] that investigated obesity, foot posture, range of motion and plantar pressure characteristics were invited to participate in this two-year longitudinal cohort study. The aim of the previous (i.e. baseline) study was to evaluate plantar loading and foot structure patterns in obese and non-obese individuals, and to determine the influence of body weight and foot structure on plantar loading. The baseline and follow-up measures were taken in 2012 and 2014, respectively, at Epworth Hospital, Victoria, Australia. Of the original 68 participants, 51 were included in this study as 17 participants were unable to attend a scheduled follow-up session. The study was approved by the Alfred Human Research Ethics Committee (HREC) and Austin Health HREC, project number 121/11. All participants provided informed consent.

### Demographic and anthropometric data

Age, gender, height and body mass were recorded at baseline and follow-up. Body weight was measured to the nearest 0.1 kg using electronic scales and height was measured to the nearest 0.1 cm using a stadiometer (with shoes, socks, and bulky clothing removed). From these data BMI was calculated in line with the baseline study [6].

### Foot pain and disability

Foot pain and disability were measured with the Manchester Foot Pain and Disability Index (MFPDI), a valid and reliable measure of foot pain and disability [11, 12]. The MFPDI consists of 19 items designed to assess four domains: functional limitation (10 items), pain intensity (5 items), personal appearance (2 items), and difficulties with work or leisure activities (2 items). Each item is preceded with the phrase, “because of pain in my feet,” and is documented as being present ‘none of the time’ (0 points), ‘on some days’ (1 point), or ‘on most/everyday’ (2 points). All scores were summed and separated into the four domains, although only functional limitation and pain intensity were used in this study. The raw scores for these domains underwent a Rasch transformation as previously described by Gijon-Nogueron et al. [13], enabling the resultant values to be treated as continuous variables in the statistical analysis. Functional limitation is graded on a 0–20 scale, whereas pain intensity is graded on a 0–10 scale.

### Plantar pressure

**Fig. 1:**
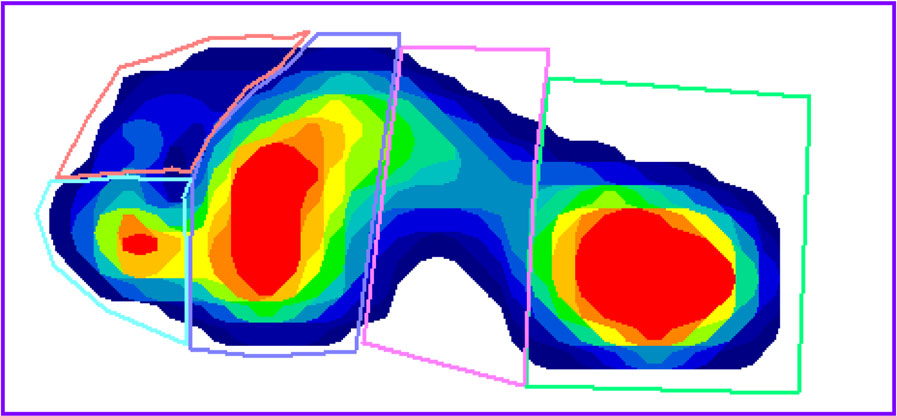
Example of individual ‘masks’, defining different regions of the foot

Dynamic plantar pressure data were collected with the MatScan® (Tekscan, USA) platform system. The platform consists of a 5 mm-thick floor mat (432 × 368 mm) incorporating 2288 resistive sensors (1.4 sensors/cm^2^) with a sampling at a rate of 40 Hz. Step calibration was performed immediately prior to each participant’s analysis. Following calibration, participants walked over the platform, which has been previously shown to have good accuracy [14] and moderate to good reliability for measuring plantar pressures in barefoot adults [15]. The MatScan® platform was positioned in the centre of a level walkway, where the participants were asked to walk barefoot in their normal gait pattern. A midgait protocol was used, whereby participants were instructed to take two steps and to then strike the platform on their third step, before continuing to walk for a further three steps. The midgait protocol has been found with few exceptions to have good to excellent reliability [16]. Data from the right foot were collected from three valid trials. Individual “masks” were manually constructed to determine plantar pressures for the whole foot and under five regions; heel, midfoot, forefoot, hallux and lesser toes, using the Research Foot software (version 6.51) at baseline and follow-up (Fig. 1). Measures of maximum force (kg), contact area (cm^2^), peak pressure (kPa) and contact time (ms) were calculated for each of the trials and an average value obtained. Contact time was used as a proxy for walking speed [17]. Change in regional peak plantar pressure was used in this study given the known association of peak plantar pressure and foot pain [18]. Mean pressure or pressure-time integral were not used in this study given the interdependence between these measures and peak plantar pressure [19, 20].

### Data analysis

All data were checked for normality prior to inferential statistical analysis. The maximum force variables (hallux and forefoot) were logarithmically transformed because they were not normally distributed. Differences between baseline and follow-up measures for anthropometry variables (height, body weight and BMI) and MFPDI subscale scores were analysed with paired-samples *t*-tests. The difference in the number of participants with foot pain at baseline and follow-up were analysed with the chi-squared test. Differences between baseline measures (age and BMI) of those who completed the study and those that failed to follow-up were analysed with Mann-Whitney *U* test, while differences in the prevalence of foot pain was analysed with the chi-squared test. Linear regression was used to test the differences between baseline and follow-up maximum force, contact area and peak pressure (adjusted for contact time). Correlations between change in body weight, change in regional peak pressure, change in foot pain intensity and functional limitation were assessed using multivariable linear regression, where unstandardised *B* coefficients were generated, adjusting for age, gender and change in contact time (where appropriate), Multivariable linear regression, adjusting for age and gender, was also used for subgroup analyses of participants whom lost more than 2 kg to provide clinical context for the association of weight loss and foot pain. Path analysis, a method used to detect hypothesised causal relationships between variables [21], was used to determine the total impact of both the direct and indirect effects of change in body weight on pressure and pain variables using standardised *β* weights. Only regions that showed significant association in the multivariable regressions were used in the path analysis. *P* values <0.05 (2-tailed) were regarded as statistically significant. All analyses were performed using the SPSS statistical package (standard version 23.0, IBM Corp, NY, USA).

## Results

### Participant characteristics

Fifty-one of 68 participants (75%), completed the two-year study. The sample had a mean (standard deviation (SD)) age of 52.6 (8.5) years. Participant characteristics are shown in Table 1. The 17 participants (14 women, 3 men) who were lost to follow-up were not significantly older, with an age median (range) of 54.9 (40.9–65.0) years versus 53.8 (34.7–67.8) years, *p* = 0.328), but did have a significantly higher baseline BMI, median (range) of 33.0 (21.4–45.2) kg/m^2^ versus 25.3 (17.6–48.1) kg/m^2^, *p =* 0.042. The prevalence of baseline foot pain was not significantly higher (58.9% versus 47.1% *χ* ^2^ = 0.706, *p* = 0.401) in those lost to follow-up. There were significantly more women than men in this study, *χ* ^2^ = 4.412, *p* = 0.036.

**Table 1:** Participant characteristics (values are the means (SD)s unless otherwise indicated)

	Baseline	Follow-up	Mean difference	95% CI	*p* value
Age	52.6 (8.5)	54.8 (8.5)			
Gender, no. women (%)	33 (65)	33 (65)			
Height, m	1.69 (0.1)	1.69 (0.1)^a^	−0.0	-0.0 to 0.0	0.145
Body mass, kg	80.3 (19.3)	82.3 (20.6)^a^	2.0	0.4 to 3.6	0.016
BMI, kg/m^2^	28.2 (6.9)	28.9 (6.9)^a^	0.6	0.1 to 1.2	0.029
MFPDI Functional limitation score	3.2 (4.5)	3.6 (5.1)^a^	0.4	−0.8 to 1.6	0.511
MFPDI Pain intensity score	1.9 (2.4)	1.9 (2.4)^a^	0.1	−0.5 to 0.6	0.784

*Abbreviations*: *SD* standard deviation, *kg* kilograms, *m* metres, *BMI* body mass index, *CI* confidence interval, *MFPDI* Manchester Foot Pain and Disability Index

^a^ *p* calculated for differences between baseline and follow-up measures analysed with paired samples t-test

### Change in body weight and BMI

Mean (SD) body weight increased from baseline to follow-up by 2.0 (5.9) kg from 80.3 (19.3), to 82.3 (20.6) kg, *p* = 0.016) as did BMI (28.2 kg/m^2^ versus 28.9 kg/m^2^, *p* = 0.029). The change in body weight ranged from −16.1 to 12.7. Twenty-five participants gained more than 2 kg, with a mean (SD) of 6.6 (3.8) kg while 11 participants lost more than 2 kg, with a mean (SD) of 5.1 (4.3) kg.

### Change in plantar pressure

The change in plantar pressure from baseline to follow-up is summarised in Table 2. The change in peak plantar pressure from baseline to follow-up ranged from −121.0 to 58.8 kPa. There were significant differences in all regions for contact area, and maximum force for whole foot, forefoot and heel before adjustment for differences in contact time. There were, however, only significant differences in the contact area of the hallux, mean (SD) 9.8 (1.7) cm^2^ to 10.6 (1.6) cm^2^, *p* = 0.017) and lesser toe regions, mean (SD) 9.5 (2.9) cm^2^ to 11.0 (2.4) cm^2^, *p* = 0.008) after adjusting for differences in contact time. The heel was the only specific region of the foot to demonstrate a significant increase in peak pressure from baseline to follow-up, mean (SD) 197 (45) to 222 (39) kPa, *p* = 0.012) after adjusting for contact time.

**Table 2:** Change in maximum force, contact area and peak plantar pressure between baseline and follow-up^a^ (values are means (SD)s unless otherwise indicated)

	Baseline	Follow-up	Mean difference	95% CI
Maximum force (kg)				
Whole foot	64.6 (19.3)	71.1 (22.1)	6.5	3.2 to 9.7
Heel	36.4 (10.5)	41.9 (12.0)	5.5	3.5 to 7.4
Midfoot	13.8 (9.1)	13.4 (8.9)	−0.4	−1.9 to 1.2
Forefoot	47.8 (13.8)	51.6 (16.1)	3.8	1.5 to 6.1
Hallux	8.0 (2.9)	8.2 (2.7)	0.2	−0.4 to 0.7
Lesser toes	4.5 (1.9)	4.5 (1.9)	0	−0.5 to 0.5
Contact area (cm^2^)				
Whole foot	109.1 (17.1)	112.7 (16.7)	3.6	2.2 to 4.9
Heel	31.0 (4.5)	32.6 (4.7)	1.6	0.9 to 2.4
Midfoot	25.6 (9.3)	23.2 (8.0)	−2.4	−3.8 to −1.1
Forefoot	47.8 (6.4)	49.2 (6.8)	1.4	0.6 to 2.3
Hallux	9.8 (1.7)	10.6 (1.6)	0.8	0.2 to 1.1*
Lesser toes	9.5 (2.9)	11.0 (2.4)	1.5	0.7 to 2.1*
Peak pressure (kPa)				
Whole foot	238 (37)	247 (42)	9	−1 to 18
Heel	197 (45)	222 (39)	25	14 to 37*
Midfoot	92 (44)	90 (45)	−2	−12 to 8
Forefoot	233 (40)	238 (46)	5	−4 to 16
Hallux	155 (42)	150 (40)	−5	−14 to 6
Lesser toes	77 (29)	74 (26)	−3	−10 to 4

*Abbreviations*: *SD* standard deviation, *kg* kilograms, *cm* ^*2*^ centimetres squared, *kPa* kilopascal, *CI* confidence interval

^a^ *p* calculated for differences between baseline and follow-up measures analysed with linear regression, adjusted for contact time

*p < 0.05

### Change in foot pain

Change in foot pain scores are detailed in Table 1. Current foot pain was reported by 24 (48%) and 28 (55%) participants at baseline and follow-up respectively. Mean (SD) functional limitation scores increased from baseline to follow-up 3.2 (4.5) points to 3.6 (5.1) points, *p* = 0.511, the change in scores ranged from −9.7 to 20.0 points. Mean (SD) foot pain intensity did not change between baseline and follow-up, but the change in scores ranged from −4.4 to 6.3 points.

### Associations between change in body weight, change in plantar pressure and change in foot pain

Multivariable associations between change in body weight, change in peak pressure and change in foot pain, after adjusting for age and gender and change in contact time (where appropriate) are summarised in Tables 3 and 4.

**Table 3:** Multivariable linear regression between change in body weight with change in regional peak plantar pressure and change in foot pain

i) Plantar pressures^a^	Unstandardised *B* coefficients (95% CI)	*p* value
Whole foot	2.151 (−2.275 to 7.186)	0.302
Heel	2.921 (−0.940 to 6.782)	0.135
Midfoot	4.648 (0.273 to 9.024)	0.038
Forefoot	2.230 (−2.231 to 6.691)	0.319
Hallux	1.444 (−3.046 to 5.935)	0.521
Lesser toes	4.303 (−1.032 to 9.638)	0.111
ii) Manchester Foot Pain and Disability Index^b^		
Pain intensity subscale	0.216 (−0.611 to 1.044)	0.601
Functional limitation subscale	0.409 (0.101 to 0.717)	0.010

Each region and pain subscale was analysed independently. The change in foot pain intensity and functional limitation units are Manchester Foot and Disability Index Rasch transformed scores (pain intensity and functional limitation domains)

*Abbreviation*: *CI* confidence interval

^a^Adjusted for age, gender and change in contact time

^b^Adjusted for age and gender

**Table 4:** Multivariable linear regression between change in peak plantar pressure and change in foot pain^a^

Region	Pain intensity	*p* value	Functional limitation	*p* value
Whole foot	1.405 (−0.237 to 3.047)	0.092	3.446 (−0.510 to 7.401)	0.086
Heel	1.831 (0.540 to 3.121)	0.006	4.054 (0.898 to 7.210)	0.013
Midfoot	1.913 (0.392 to 3.434)	0.015	4.505 (0.825 to 8.186)	0.018
Forefoot	1.450 (−0.087 to 2.987)	0.064	3.184 (−0.548 to 6.915)	0.093
Hallux	−0.352 (−1.945 to 1.241)	0.672	1.120 (−2.716 to 4.956)	0.560
Lesser toes	0.623 (−1.309 to 2.556)	0.520	2.530 (−2.092 to 7.151)	0.276

Each region and pain subscale was analysed independently. Change in foot pain intensity and functional limitation units are Manchester Foot and Disability Index Rasch transformed scores (pain intensity and functional limitation domains)

Values are unstandardised *B* coefficients (95% confidence interval)

^a^Adjusted for age, gender and change in contact time

As body weight increased, peak pressure increased in all regions, however the midfoot was the only region to show significant, positive correlation with body weight in multivariable regression (*B* = 4.648, 95% CI 0.273 to 9.024, *p* = 0.038). There was also a significant, positive correlation between change in body weight and change in functional limitation (*B* = 0.409, 95% CI 0.101 to 0.717, *p* = 0.010), but not pain intensity (*B* = 0.216, 95% CI -0.611 to 1.044, *p* = 0.601).

There were positive, significant correlations between changes in heel (*B* = 1.831, 95% CI 0.540 to 3.121, *p* = 0.006) and midfoot (*B* = 1.913, 95% CI 0.392 to 3.434, *p* = 0.015) peak pressure and change in foot pain intensity, and a significant, positive correlation between changes in heel (*B* = 4.054, 95% CI 0.898 to 7.210, *p* = 0.013) and midfoot (*B* = 4.505, 95% CI 0.825 to 8.186, *p* = 0.018) peak pressure and change in functional limitation.

Of the 11 participants whom lost more than 2 kg, there was a significant positive correlation between change in weight and change in functional limitation (*B* = 0.654, 95% CI 0.174 to 1.134, *p* = 0.015), and there was a non-significant positive correlation between change in weight and change in pain intensity, (*B* = 0.274, 95% CI -0.009 to 0.556, *p* = 0.056).

### Path analysis

**Fig. 2:**
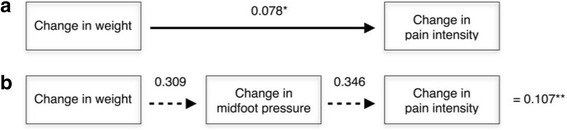
Calculation of direct and indirect effects of change in body weight on change in pain intensity. Values are standardised *β* coefficients: (**a**) direct effect of change in body weight on foot pain intensity, (**b**) indirect effect of change in body weight, mediated by change in midfoot pressure. (*) Direct effect, (**) Indirect effect. The total effect of change in body weight on foot pain intensity is therefore the sum of the direct and indirect effects, i.e. total impact is 0.078 + 0.107 = 0.185

**Fig. 3:**
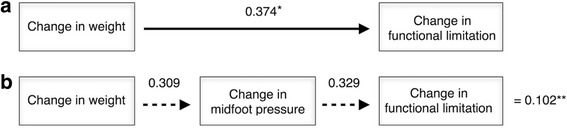
Calculation of direct and indirect effects of change in body weight on change in functional limitation. Values are standardised *β* coefficients: (**a**) direct effect of change in body weight on functional limitation, (**b**) indirect effect of change in body weight, mediated by change in midfoot pressure. (*) Direct effect, (**) Indirect effect. The total effect of change in body weight on functional limitation is therefore the sum of the direct and indirect effects, i.e. total impact is 0.374 + 0.102 = 0.476

Results of the path analysis are shown in Figs. 2 and 3. For pain intensity, there was a small (*β* = 0.078) direct effect of change in body weight, but a larger indirect effect with change in midfoot pressure as a mediator variable (*β* = 0.107). For functional limitation, change in body weight had a larger direct (*β* = 0.374) than indirect (*β* = 0.102) effect with change in midfoot pressure as a mediator variable. The total effect of change in body weight (i.e. the combined direct and indirect effects) was smaller for pain intensity (*β* = 0.185) than functional limitation (*β* = 0.476).

## Discussion

This study is the first to examine the effect of increasing body weight on plantar pressures and foot pain using a prospective study design. Such a design allows for temporal inferences to be made. There were significant associations between change in body weight, change in midfoot plantar pressure and change in functional limitation. Change in heel plantar pressure was significantly associated with a change in functional limitation, but not a change in body weight. Path analysis indicated that the effect of increasing body weight on foot related functional limitation may be mediated by increased plantar pressure in the midfoot, supporting a significant biomechanical effect. These findings suggest that as body weight increases, foot pain increases, and that the midfoot may be the most vulnerable site for pressure-related pain.

Change in body mass was not significantly associated with change in foot pain intensity, but there were significant, positive correlations between change in foot pain intensity and change in both heel and midfoot peak pressure. While there were statistically significant increases in contact area of the hallux and lesser toes from baseline to follow-up, following adjustment for differences in contact time, these are likely to be of questionable clinical significance given the lack of significant increases in peak pressures in these regions. The heel was the only site to increases in peak pressures following adjustment for differences in contact time. This suggests that the foot may be able to modulate force and contact area to reduce peak pressure, however given the heel is usually the first region to strike the ground in normal gait, [22] this region may be less efficient in increasing contact area. Previous studies investigating the effect of increasing body weight on plantar pressure have traditionally used weighted backpacks or vests [23–25], and therefore, have measured the instantaneous effects of increased body weight, and not weight that is physiologically gained over time. Previous studies have also used asymptomatic volunteers, which may not reflect how plantar pressures change with not only body mass gain, but also with foot pain. In contrast, our study examined the effect of increasing body weight on plantar pressures over time and measured this in the context of foot pain intensity and functional limitation.

The results of this study provide evidence to support the assertion that increases in peak plantar pressure are associated with foot pain and disability. Given that pain intensity and functional limitation increased as peak pressure under the midfoot and heel regions increased, these regions may be most at risk from increasing body weight. Furthermore, the significant positive correlation with body weight and peak pressure under the midfoot, but not other regions, is suggestive that the mechanical link between increased body weight, increased plantar pressure and pain is focused in this region in particular. The positive association with plantar pressure and pain in this study are inconsistent with a recent study that found people with prolonged plantar heel pain paradoxically had reduced peak pressure in this region [26]. The authors suggested that this may be an offloading mechanism, which could be initiated as pain increases beyond tolerable levels. That is, people with plantar heel pain adopt an antalgic gait pattern to reduce resultant pressure from the ground being applied to the painful heel when walking. The association between increases in plantar pressure and foot pain observed in our study may reflect less disabling foot pain not yet requiring gait alterations to offload the painful region.

While a change in foot pain intensity was not significantly associated with a change in body weight, studies have found body composition, as opposed to body weight alone, may be more strongly associated with pain. An increase in fat mass, rather than fat-free mass, is the main component of body mass that contributes to foot pain [10, 27] and likely does so via metabolic as opposed to mechanical pathways. The association between body weight and functional limitation may indicate that increasing body weight affects the ability to undertake daily activities more so than increasing the intensity of pain.

This study should be considered in light of some limitations. The site of foot pain was not recorded and we cannot, therefore, draw conclusions as to whether the region of increased plantar pressure corresponded to the region of pain. Differences in pressure between those with bilateral or unilateral foot pain was also not explored. There was a relatively small sample size, and the modest increase in body mass over the two-year period may also limit extrapolations for larger gains in body weight. A change in body weight of greater than 5% is considered clinically relevant, whereas our cohort increased by only 2.5% [28]. Those who took part in this study tended be younger and have a lower BMI than those lost to follow-up. Thus, our results are generalisable to this population only, which may also reduce the power of the study since the spectrum of obesity and foot pain was reduced. Minimal important differences for the MFPDI domains scores are not available [29] and therefore the clinical importance of changes in these scores cannot be determined.

The main clinical implication of this study is that higher peak pressures in the heel and midfoot are most strongly related to pain intensity and functional limitation as body weight increases. The midfoot may, therefore, be the most susceptible region to developing pain following weight gain and interventions that reduce pressure in this region may reduce foot pain. Moreover, the 11 participants that lost more than 2 kg had a significant correlation between change in functional limitation and change in weight, this provides temporal evidence that weight loss is associated with reduced foot pain, but studies involving larger samples and clinical trials with directed weight loss interventions are needed. Indeed, future research is required to determine whether interventions designed to normalise or decrease plantar pressures can reduce foot symptoms over time.

## Conclusion

Increasing body weight is associated with increasing midfoot plantar pressure and foot-related functional limitation over a two-year period, while changes in midfoot and heel plantar pressures are associated with changes in foot pain intensity. These findings suggest that as body weight and plantar pressure increase, foot pain increases, and that the midfoot may be the most vulnerable site for pressure-related pain.

## Abbreviations

BMI: body mass index
CI: confidence interval
cm: centimetre
HREC: Human Research Ethics Committee
Hz: Hertz
IBM: International Business Machines Corporation
kg: kilogram
kPa: kilopascal
MFPDI: Manchester Foot Pain and Disability Index
ms: millisecond
NY: New York
SD: standard deviation
SPSS: Statistical Package for the Social Sciences
USA: United States of America
